# Monitoring the Changing Patterns in Perceived Learning Effort, Stress, and Sleep Quality during the Sports Training Period in Elite Collegiate Triathletes: A Preliminary Research

**DOI:** 10.3390/ijerph19084899

**Published:** 2022-04-18

**Authors:** Yi-Hung Liao, Chih-Kai Hsu, Chen-Chan Wei, Tsung-Chieh Yang, Yu-Chi Kuo, Li-Chen Lee, Li-Ju Lin, Chung-Yu Chen

**Affiliations:** 1 Department of Exercise and Health Science, National Taipei University of Nursing and Health Sciences, Taipei 112, Taiwan; yihungliao.henry@gmail.com (Y.-H.L.); lout0629@gmail.com (C.-K.H.); yuchi.kuo@gmail.com (Y.-C.K.); 2 Department of Aquatic Sports, University of Taipei, Taipei 111, Taiwan; tom911072@gmail.com; 3 Dodo Bird Exotic Animal Hospital, Hsinchu 300, Taiwan; centromerevm@gmail.com; 4 Office of Physical Education, Shih-Hsin University, Taipei 116, Taiwan; lilychen@mail.shu.edu.tw; 5 Smart Healthcare Interdisciplinary College, National Taipei University of Nursing and Health Sciences, Taipei 112, Taiwan; 6 Department of Exercise and Health Sciences, University of Taipei, Taipei 111, Taiwan

**Keywords:** athletes, fatigue, mood, academic learning states

## Abstract

Background: Few studies have examined the mental profiles and academic status of collegiate triathletes during training/competitive periods. We evaluated the changes in sleep quality, physical fatigue, emotional state, and academic stress among collegiate triathletes across training periods. Methods: Thirteen collegiate triathletes (19–26 years old) were recruited in this study. Mood state, sleep quality, degree of daytime sleepiness, subjective fatigue, and academic learning states were measured during the following five training periods: before national competitions for 3 months (3M-Pre Comp), 2 months (2M-Pre Comp), 1 month (1M-Pre Comp), 2 weeks (2wk-Pre Comp), and national competition (Comp) according to their academic/training schedule. Results: The academic stress index in 1M-Pre Comp (Final exam) was significantly higher than that in 3M-Pre Comp in these triathletes. No markedly significant differences were observed in overall mood state, sleep quality, individual degree of sleepiness, and fatigue among these five periods. However, the profiles mood state scale (POMS)-fatigue and -anger were lower in 2wk-Pre Comp than that in 1M-Pre com. The POMS-tension score in Comp was significantly higher than that in 3M-Pre Comp and 2M-Pre Comp. POMS-depression in Comp was lower than that in 1M-Pre Comp. Conclusion: We found that training volume was highest one month before a competition, and the academic stress is greatest during their final term exam period (1M-Pre Comp). After comprehensive assessment through analyzing POMS, PSQI, ESS, and personal fatigue (CIS), we found that the collegiate triathletes exhibited healthy emotional and sleep states (PSQI score < 5) across each training period, and our results suggest that these elite collegiate triathletes had proficient self-discipline, time management, and mental adjustment skills.

## 1. Introduction

A triathlon is a competitive sporting event that combines swimming, cycling, and running. Competition distances vary in different triathlon formats [1]. The biggest difference between triathlons and other sporting events is that triathletes spend more time training than other athletes. Each of the three disciplines requires separate extensive training. Consequently, these athletes may be under greater stress physically, psychologically, and socially [2]. Triathlon training must be tailored to an individual’s needs and condition. Training periodization and a daily schedule are crucial [2]. Training periodization is a means of helping athletes reach their physical and result goals and involves the scheduling of long, moderate, and short training cycles and sessions [3]. Periodization can help triathletes achieve peak levels of regulation and maximum performance during the most important event of the year. Athletes and their coaches should thus pay attention to the type of physical training adaptation required and use the necessary training or skills to achieve a particular adaptive response to physical training [4].

Triathlons are organized worldwide and year-round and comprise both events for elite athletes and leisure competitions. Long duration training that is too intense, combined with insufficient recovery time, can reverse the many positive physiological adjustments associated with training adaptation, resulting in overtraining [5]. Training intensity affects an athlete’s body and psychology to certain levels, and appropriate overtraining can induce athletic performance enhancement. However, an excessive training intensity combined with other stress factors may result in overtraining syndrome (OTS) [6]. Researchers widely believe that endurance athletes are at higher risk of OTS due to the prolonged and massive physical and psychological stress they are under due to, for example, training and competing [7]. Clinical diagnosis of OTS is made when athletes exhibit fatigue, mood changes, sleep deprivation, declining athletic performance, and increasing frequency of injury and pain [7]. However, very few studies have investigated the physical and mental stress on triathletes resulting from training and competing.

School sports events are sports competitions primarily for university athletes and include triathlons. From the perspective of specialized sports training, the literature indicates that sufficient research has been conducted into different training period levels, athletic performance, and physical and psychological stress. For example, studies have compared professional elite athletes [1,8,9] and investigated sex differences in athletic performance [10], athletic performance in specific disciplines [8,10], and changes in physiological traits throughout a season [11,12,13]. Student athletes, especially elite athletes, need to deal with frequent training and high training intensity and volume in their periodic training schedule. However, they must also spend considerable time and energy maintaining their academic performance [14]. Due to the diverse and massive training intensity for triathlons in particular, we believe that the external and internal loadings on student athletes are not smaller than the physical and mental stress experienced by professional athletes in any other competitive sport. According to surveys, collegiate athletes often experience problems with sleep, emotions, and fatigue [15,16,17]. Although numerous factors can affect the overall performance of triathletes, research into the effect of specific training factors on burnout among triathletes is currently lacking [11,18]. Crucially, the extreme pressure exerted by a heavy learning workload and intense athletic training at this stage can disrupt student athletes’ normal physiological and psychological development. Comprehensive assessments of triathletes’ mood, sleep, internal and external loadings, fatigue, and athletic performance for different disciplines and training cycles are relatively lacking. Therefore, in the field of sports medicine, more research into the influence of the type of training periodization on students who engage in long and intensive training is necessary.

We hypothesized that the perceived learning effort, mental stress status, and sleep quality would be negative impacted by the increasing training intensity across different periodic training periods in these elite collegiate triathletes. This study evaluated the differences in training conditions, sleep quality, emotional status, and academic pressure among collegiate triathletes with different training cycles. The possible factors affecting these variables were investigated. The evaluation results are used to explore the physical and psychological changes in collegiate triathletes. Multiple questionnaires through periodic survey were used to assess the physical and mental state of student triathletes across different training periods, as well as provide a comprehensive assessment of their academic stress. Therefore, the findings provide coaches and supervisors in sports sciences with reference data when scheduling training for collegiate triathletes and can assist student athletes to avoid injuries due to intense training and overtraining for competitions.

## 2. Materials and Methods

### 2.1. Participants and Ethical Statement

The participants in this study were 13 collegiate triathletes (5 females, 8 males) between the ages of 19–26 years. The anthropometric profiles of the participants are shown in Table 1. All participants had fixed formulated annual training schedules under the supervision of their team coach. During this study, the researchers did not change the coaches’ training schedule. Participants that met the following conditions were excluded: (1) inability to comply with the training schedule; (2) severe skeletal or muscular injury within the preceding 3 months; (3) mental illness; (4) heart disease, diabetes, or other metabolic diseases. The researchers explained the experimental process to the collegiate triathlon coaches and then announced the recruitment through posters. Before the study officially started, the researchers explained the research process and precautions to all participants. Participants were required to sign informed consent forms. This study was performed according to the last version of the Helsinki Declaration and approved by the Institutional Review Board (IRB) at the University of Taipei (IRB-2018-066).

### 2.2. Study Design and Procedure

**Figure 1:**
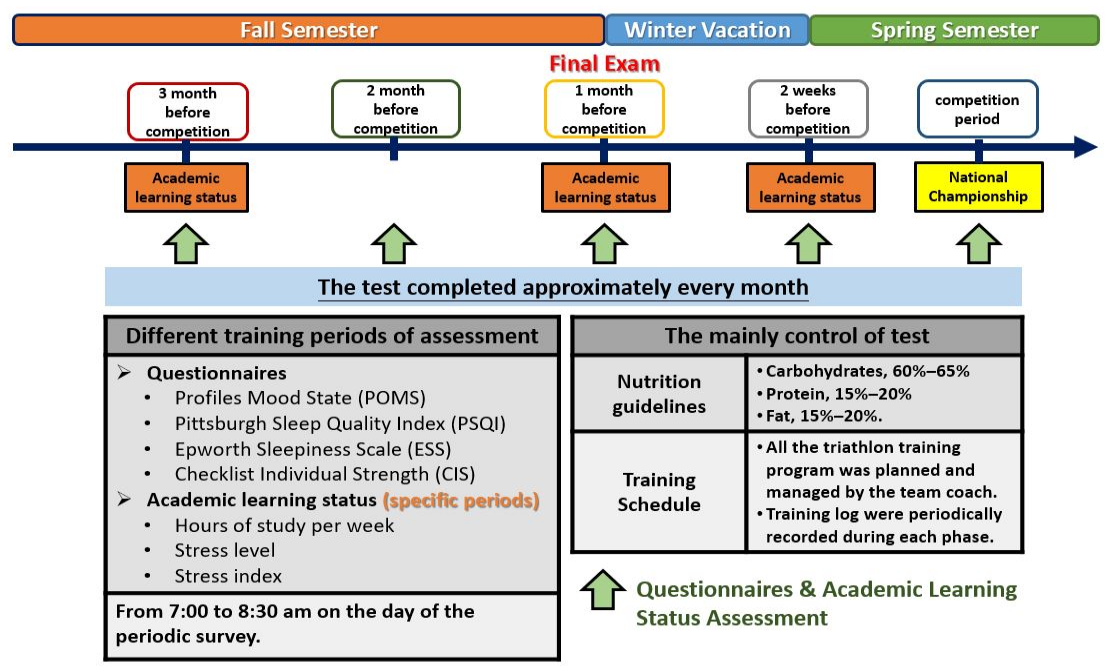
The collegiate triathlete periodic training program detailed procedure and time frame.

To minimize any effect exerted by the students’ previous training, the coaches avoided scheduling intense exercise and resistance training for 2 days prior to physical performance tests. The questionnaire comprised a sleep quality scale, mood scale, daytime sleepiness scale, and fatigue questionnaire. From 7:00 to 8:30 am on the day of the periodic survey, participants completed four different questionnaires after measuring anthropometric test, including the Profiles Mood State (POMS) scale for mood state evaluation, Pittsburgh Sleep Quality Index (PSQI) scale for sleep quality evaluation, Checklist individual strength (CIS) for subjective fatigue, and Epworth Sleepiness Scale (ESS) scale for daytime sleepiness evaluation. To ensure adequate dietary habit control throughout the training period, we gave the athletes detailed nutritional guidelines. Based on their individual daily energy demands and training period, the participants were instructed to consume the following food groups in these proportions: carbohydrates, 60–65%; protein, 15–20%; and fat, 15–20%, according to the previous study focusing on triathletes [19]. The detailed procedure and timeframe of this study are illustrated in Figure 1.

### 2.3. Anthropometric Measurements

The participants were requested to avoid any form of exercise for 2 days before the anthropometric measurements and fast for 10 h before the anthropometric test. All instruments were calibrated according to the manufacturers’ directions before the testing. Height was measured using a Dong Sahn Jenix height scale (Seoul, Korea). Weight, body fat percentage, fat-free weight, body mass index, and other anthropometrics were measured using the OMRON HBF-371 body composition monitor (Kyoto, Japan) based on the bioelectrical impedance method. The participants were requested to wear the same light clothing to all measurement sessions to ensure consistency across their pretest and posttest bioelectrical impedance analysis (BIA) measurements. Further, all participants were instructed to prohibit from drinking water and any fluids containing caffeine 2 h prior to the BIA assessment to minimize possible interference and ensure consistency. To ensure the reliability of the BIA measurements, the analysis was conducted with the participants standing in their bare feet on the electrode plate with their hands on the electrode handles.

### 2.4. Triathletes Periodic Training Program

**Figure 2:**
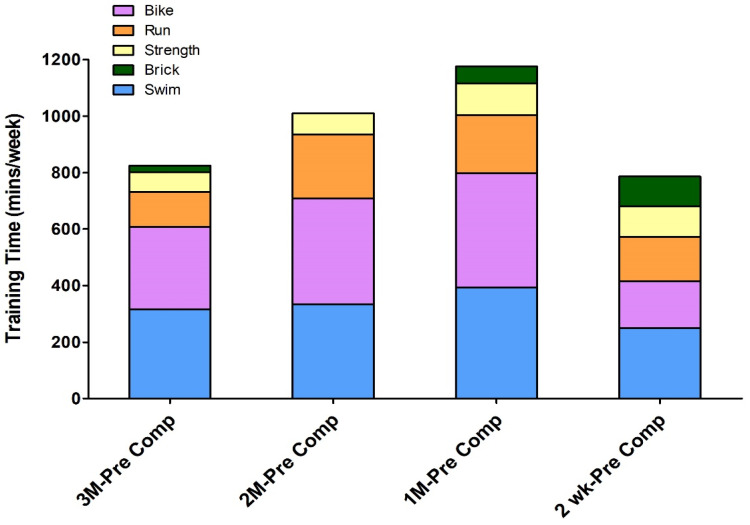
Training schedule for the different training periods. 3M-Pre Comp: 3 months before competition, 2M-Pre Comp: 2 months before competition, 1M-Pre Comp: 1 month before competition, 2wk-Pre Comp: 2 weeks before competition.

The triathletes were also asked to maintain their training logs during their periodic training periods, and the training log was the training diary recorded by their team coach to record individual athletes’ training type, frequency, intensity, and training feedback. These logs were used to calculate their training intensity for specific sports disciplines. Figure 2 shows the training program during the different training periods. The triathlon training consisted of running, swimming, cycling, and strength/brick training. All participating athletes were performing their regular training under the coach’s supervision during the periodic training program. The weekly training time for the four training periods was 824 min (3 months before competition, 3M-Pre Comp), 1010 min (2 months before competition, 2M-Pre Comp), 1175 min (1 month before competition, 1M-Pre Comp), and 786 min (2 weeks before competition, 2wk-Pre Comp). During the data collection period, it can be seen that the majority of training hours were spent on swimming and cycling, and the training duration for these two sports disciplines was about 1:1, followed by running and then weight training. While the brick training took up the least amount of total training, it can still be seen that the training time proportion for brick training increased 2 weeks before the national competition events.

### 2.5. Perceived Learning Effort and Study Time

Participants were asked to rate their level of perceived learning effort on a Likert scale of 1 (very easy) to 10 (extremely stressful) for their regular subjective studying effort and academic preparation during their specific training periods. In addition, participants were asked to provide the average number of study hours per week during the specific training period for the perceived learning work effort assessment. Banister et al. calculated the training impulse (TRIMP) by multiplying the exercise duration by the exercise intensity, which mainly reflects the overall exercise volume during exercise [20]. Our investigation applied this concept into consideration and designed an academic stress index calculated by multiplying perceived learning effort by studying duration to represent overall academic stress/volume across different training periods. In brief, the triathlete academic stress index during each training period was calculated using the product of the subjective perceived learning effort level during their academic activities and the average number of hours of study per week for that training cycle.

### 2.6. Profiles Mood State Questionnaire (POMS)

The POMS was used to measure the emotional state of the athletes under specific training and competition conditions. The POMS questionnaire was developed by McNair et al. and comprises 37 questions covering seven elements [21]. The elements vigor (7) and self-esteem (5) represent positive moods, whereas the elements confusion (7), fatigue (6), anger (5), tension (4), and depression (3) represent negative moods. Each question was graded on a 5-point Likert scale from 0 (not at all) to 4 (extremely), and the score for each item was directly added to the total. When answering each question, the participants responded to the question “How have you felt for the past 2 weeks (including today)?”.

### 2.7. Pittsburgh Sleep Quality Index (PSQI)

The Chinese version of the PSQI scale, as translated by Tsai et al., was used in this study [22]. The PSQI scale has nine questions covering seven dimensions: sleep quality, sleep latency, sleep time, habitual sleep efficiency, sleep disorders, sleep medication use, and daytime dysfunction. Each dimension is worth 0–3 points; the maximum number of points is 21. A higher PSQI score indicates poorer sleep quality. Typical scores are less than or equal to 5, signifying favorable sleep quality. The participants completed the scale according to their sleep during the previous month. The Cronbach’s α for the scale is 0.8332. The Chinese PSQI validity was verified using the PSQI scale tests. The questionnaire’s accuracy, sensitivity, and specificity were found to be 88.5%, 89.6%, and 86.5%, respectively (κ = 0.75; *p* < 0.001), demonstrating that the Chinese PSQI scale has satisfactory reliability and validity.

### 2.8. Epworth Sleepiness Scale

The Epworth Sleepiness Scale (ESS) is a questionnaire scale used to assess the degree of daytime sleepiness of participants [23]. The ESS is a subjective evaluating scale that allows participants to self-assess the probability of dozing in eight different situations (e.g., sitting and reading, watching television, etc.) on a scale ranging from 0 (no probability of dozing) to 3 (high probability of dozing). The scores were mainly calculated by summing the scores of the eight questions and the total score represented their daytime sleepiness. A score in the range of 0–9 was considered normal, while a score in the range of 10–24 indicated excessive drowsiness.

### 2.9. Checklist Individual Strength (CIS)

The Checklist Individual Strength (CIS) questionnaire was used to assess the subjective fatigue level of triathletes at each training period. The CIS questionnaire consists of 20 items, and each question is scored on a 7-point Likert scale. The total score for the test was obtained by summing the item scores. These 20 items were spread over four subscales: fatigue severity, which measures (i) subjective fatigue (8 items); (ii) concentration, which measures attention problems (5 items); (iii) motivation, which measures decreased motivation (4 items); and (iv) physical activity, which measures decreased activity (3 items). In the CIS questionnaire, some items were reverse-scored for more precise assessment. CIS exhibits reliable internal consistency (α = 0.84–0.95) [24].

### 2.10. Statistical Analysis

SPSS 16.0 (Chicago, IL, USA) and GraphPad Prism 5.0 (La Jolla, CA, USA) were used to analyze and graph the data, respectively. SPSS 16.0 was employed to perform the Shapiro–Wilk normality test to analyze the normality of the variables of interest. One-way analysis of variance and repeated measures were used to compare changes in academic status, emotional status, subjective fatigue, daytime sleepiness, and sleep quality during the overall training and competition periods. All data were expressed as the mean ± standard error of the mean, and the level of significance in all comparisons was set as 0.05 (*p* < 0.05).

## 3. Results

### 3.1. Effects of Different Training Periods on Academic Stress Status of Triathletes

**Figure 3:**
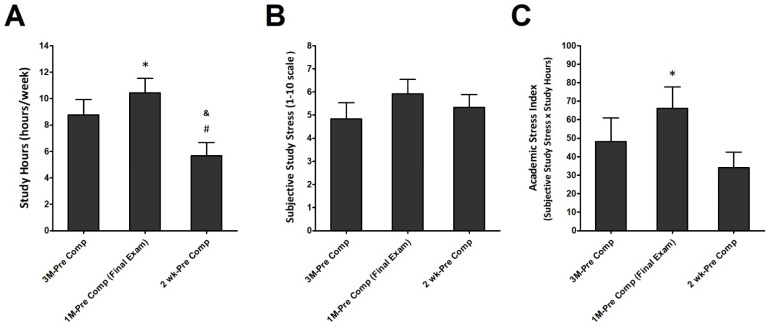
Subjective academic learning effort and stress status. (**A**) Study hours, (**B**) subjective study stress, and (**C**) academic stress index (assessed by multiplying study stress and study hours) were measured and collected at three training period. Data are represented as Mean ± S.E.M. * denotes significant difference between 3M-Pre Comp and 1M-Pre Comp (Final exam) (*p* < 0.05). # denotes significant difference between 3M-Pre Comp and 2wk-Pre Comp (*p* < 0.05). & denotes significant difference between 1M-Pre Comp (Final exam) and 2wk-Pre Comp (*p* < 0.05). See the legend of Figure 2 for abbreviations.

Figure 3 presented the athlete’s academic learning status results during different training periods. Figure 3A displays the subjective weekly study hours. The weekly study hours were significantly higher in 1M-Pre Comp (Final exam) compared with 3M-Pre Comp. However, 2 wk-Pre Comp presented significantly lower weekly study hours compared with 3M-Pre Comp and 1M-Pre Comp (Final exam) (*p* < 0.05; η^2^ = 0.226). There were no differences among the three phases for subjective study stress in these athletes (Figure 3B). The academic stress index in 1M-Pre Comp (Final exam) was significantly higher than that in 3M-Pre Comp in this population (Figure 3C) (*p* < 0.05; η^2^ = 0.109).

### 3.2. Effects of Different Training Periods on Mood States in Triathletes

**Figure 4:**
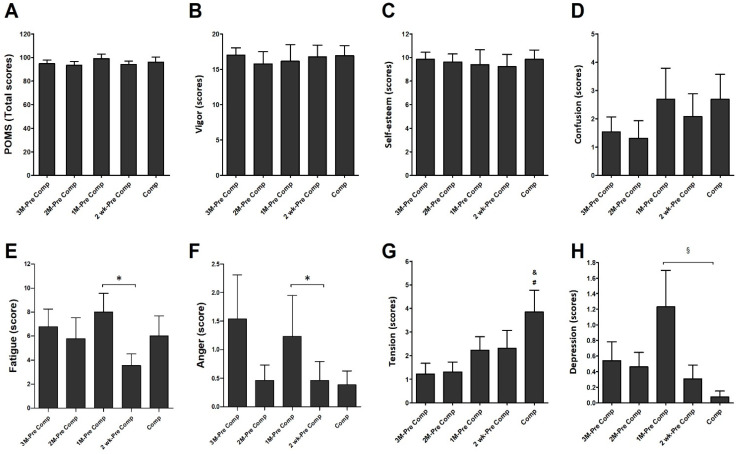
Profile of Mood state. (**A**) Total scores of POMS, (**B**) Vigor of POMS, (**C**) Self-esteem of POMS, (**D**) Confusion of POMS, (**E**) Fatigue of POMS, (**F**) Anger of POMS, (**G**) Tension of POMS, and (**H**) Depression of POMS were measured in five training periods. Data are represented as Mean ± S.E.M. * denotes significant difference between 1M-Pre Comp and 2wk-Pre Comp (*p* < 0.05). & denotes significant difference between 3M-Pre Comp and Comp (*p* < 0.05). # denotes significant difference between 2M-Pre Comp and Comp (*p* < 0.05). § denotes significant difference between 1M-Pre Comp and Comp (*p* < 0.05). See the legend of Figure 2 for abbreviations.

POMS questionnaire was used to assess the mood state and is shown in Figure 4. There were no significant differences in overall mood states (Figure 4A), anger (Figure 4B), self-esteem (Figure 4C), and confusion (Figure 4D) among each period in these athletes. However, the fatigue (Figure 4E) and anger (Figure 4F) scores were significantly lower in the 2 wk-Pre comp compared with the 1M-Pre comp (*p* < 0.05; fatigue: η^2^ = 0.022; anger: η^2^ = 0.065). The tension scores were significantly higher in Comp than in 3M-Pre Comp (3 month before competition) and 2M-Pre Comp (Figure 4G) (*p* < 0.05; η^2^ = 0.148). The level of depression during competition period (Comp) was significantly lower than in 1M-Pre Comp (Figure 4H) (*p* < 0.05; η^2^ = 0.152).

### 3.3. Effects of Different Training Periods on Degree of Fatigue and Sleepiness in Triathletes

**Figure 5:**
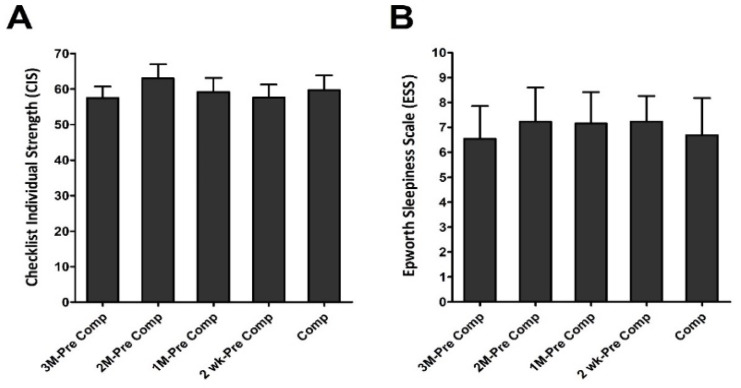
Individual degree of fatigue and sleepiness. Checklist individual strength (**A**) and Epworth sleepiness scales (**B**) were collected at five training periods. Data are represented as Mean ± S.E.M. See the legend of Figure 2 for abbreviations.

The individual degrees of fatigue and sleepiness assessed by Checklist Individual Strength (CIS) questionnaire and Epworth Sleepiness scale (ESS), respectively, are shown in Figure 5. No significant difference in individual fatigue was observed among these five different training phases (Figure 5A). Additionally, we did not observe any significant differences in degree of sleepiness among the five different training periods in this population (Figure 5B).

### 3.4. Effects of Different Training Periods on Sleep Quality in Triathletes

**Figure 6:**
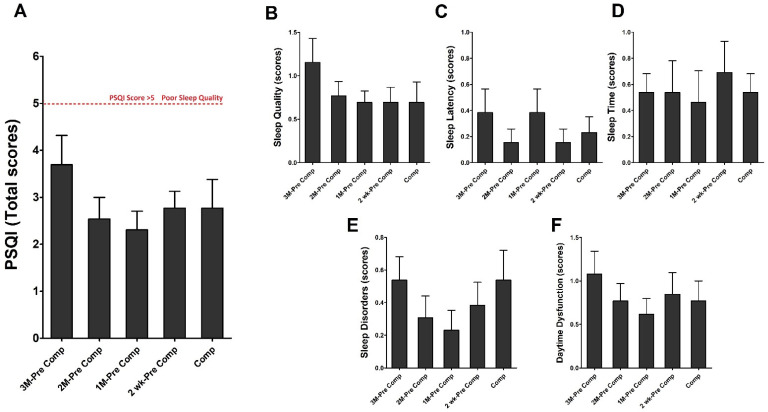
Pittsburgh Sleep Quality Index (PSQI). (**A**) Total PSQI, (**B**) PSQI Sleep quality, (**C**) PSQI Sleep latency, (**D**) PSQI Sleep time, (**E**) PSQI Sleep disorders, and (**F**) PSQI Daytime dysfunction scores were determined in five training periods. Data were represented as Mean ± S.E.M. See the legend of Figure 2 for abbreviations.

Using the PSQI questionnaire to evaluate sleep quality is shown in Figure 6. The overall sleep quality scores in these triathletes for the five different training periods (3M-Pre Comp, 2M-Pre Comp, 1M-Pre Comp, 2wk-Pre Comp, Comp) were not significantly different (Figure 6A) (*p* > 0.05). In addition, there were no significant differences among periods in subjective sleep quality (Figure 6B), sleep latency (Figure 6C), sleep time (Figure 6D), sleep disorders (Figure 6E), and daytime dysfunction (Figure 6F) (*p* > 0.05).

## 4. Discussion

The major finding of this study was that the total number of hours that the collegiate triathletes spent in training sessions—which comprised running, swimming, cycling, weight training, and brick training—gradually increased initially. The total training amount then gradually decreased after reaching a peak 1 month before a competition. Furthermore, more training hours were spent in swimming and cycling than in the other training types. The academic stress index, which was assessed by multiplying study stress and study hours, exhibited that these athletes experienced both greater academic and sports training intensity one-month before national competition events (final term exam period). The ESS and personal fatigue scale results revealed no significant differences between training months during the investigation period. The PSQI results showed no significant difference in the total score for each month (each month’s score was less than 5 points), nor any significant difference for the PSQI scale subindexes, namely, subjective sleep quality, sleep latency, habitual sleep efficiency, total sleep hours, sleep disorders, sleep medication use, and daytime dysfunction. The POMS scale results revealed that the total emotional score did not differ significantly between the monitored months. However, the individual POMS scores indicated that the tension index was higher during competition months than other training months. The depression index was highest 1 month before a competition and significantly higher than that in the competition period. The fatigue index was highest 1 month before a competition and, at this time point, it was significantly higher than that 2 weeks before the competition period. No significant differences were discovered in the other POMS assessment indexes—vigor, self-esteem, and anger. Despite the extensive amount of training that the participants underwent during the group training 1 month before a competition, the personal fatigue index scores were not significantly higher during this time.

Triathlon training plans involve running, swimming, cycling, and weight training. In keeping with the basic concepts of the three triathlon stages, the training for each discipline was incorporated into the following annual competition periods: off season, preseason, and competition season [4]. Researchers have demonstrated that in nonelite triathletes, training for 8–10 h total per week (5–6 h cycling and 3–4 h running per week) resulted in the lowest likelihood of injury [18]. Furthermore, during the off season, triathletes focus on mental and physical rest and emphasize resistance training to improve their athletic performance and prevent injury through improving muscle strength [2]. To improve aerobic endurance, training plans must be designed to strengthen the respiratory, cardiovascular, and musculoskeletal system functions, such as through physical strength training to improve athletic performance in specific disciplines [4]. The average periodic training hours for the collegiate triathletes participating in this study were 4–6.5 h of swimming, 3–7 h of cycling, and 2–4 h of running, which are largely equivalent to the hours recommended. The training schedule also included weight training each month, the average duration of which was 1.5 h. One study demonstrated that the triathletes’ training period and daily schedule are also critical [2]. As competition season approaches, training periods begin with high-volume, low-intensity training and progresses to low-volume, high-intensity training [4]. The present study discovered that the number of brick training hours increased with the approach of national level competitions. The number of hours for other types of training also increased but peaked 1 month before the competition before decreasing. This demonstrated that the training schedules for collegiate triathletes conform to periodic training patterns. However, in terms of athletic intensity, the physical activity measurements performed in this study did not indicate any significant differences between periods. As the rate of perceived exertion (RPE) and session RPE (sRPE) were not measured, this study was unable to fully understand training intensity. Future studies should use RPE and sRPE to analyze differences in intensity between training periods in greater detail.

Training intensity monitoring is necessary for understanding an individual’s response to training stimuli and assessing the levels of fatigue during different periods. The present study discovered that training intensity and training tension were significantly greater during intensive training periods and were significantly positively correlated with the total fatigue score. This indicated that subjective fatigue surveys are a sensitive tool and can be used to perceive changes in training intensity [5]. The Checklist Individual Strength subjective fatigue scale was used in this study and revealed a lack of significant differences in personal fatigue between each training month during the study period. Although the athletes had periodic training schedules, the study results demonstrated that changes to the training volume each month did not significantly influence the fatigue felt by the athletes. According to our results, periodic training schedules do not create significant changes in collegiate triathletes’ subjective fatigue. Another possible explanation is that triathletes undergo massive amounts of training in each training period; these athletes are thus fatigued for long periods of time and do not receive sufficient relief from fatigue.

Previous evidence has revealed that the athletes’ sleep time and quality dropped after their training intensity increased (+30%), demonstrating an inverse correlation between training intensity and sleep time and quality [25]. Athletes are often unable to obtain sufficient sleep due to training, competing, competition scheduling, travel, stress, and other training intensities and times or due to overtraining. Many other factors may also lead to insufficient sleep in overtrained athletes [25,26]. For example, academic pressure or work burdens and even psychological pressure can negatively affect athletes’ energy [17]. The PSQI assessments of triathletes’ sleep during this study revealed that the total scores were lower than 4 points and the assessments for each period were not significantly different. The subindexes—sleep quality, sleep latency, habitual sleep efficiency, total sleep time, sleep disorders, sleep medication use, and daytime dysfunction—did not exhibit significant differences, demonstrating that collegiate triathletes commonly experienced good and long sleep. Furthermore, we discovered that the athletes trained the most but had the lowest sleep quality index scores at 1 month before a competition, indicating that the collegiate triathletes had higher sleep quality when they were training more. However, this is inconsistent with studies reporting that an increase in training intensity leads to lower sleep quality and total amount of sleep [25,26]. We noted that 1 month before a competition was during the winter holiday break, when the athletes’ academic pressure was relatively low; consequently, the athletes were better able to fully recover (including sleep) from high-intensity training during this period. The present results also revealed that the competition season and training schedule for student athletes may need to be planned with comprehensive consideration of their school semester schedules to ensure they have time to rest and recover while balancing academic and training demands.

Interestingly, during the months with the greatest training volume, only the POMS scale depression and fatigue scores increased. The personal fatigue scale scores did not increase. This was possible due to the collegiate athletes’ peak training period coinciding with the winter holiday break—when the athletes could obtain sufficient rest. Therefore, the high POMS fatigue score may have been the result of the athlete’s mental state, which could have been affected by psychological factors such as the athlete’s own self-demands and their coach’s demands. Athlete’s fatigue may thus originate from psychological pressure while training rather than from increased physical activity. Training hours were consistent with the durations suggested in the literature, and the few differences may have been because the literature suggestions were for nonelite athletes. The study participants were collegiate elite athletes, and no severe sports injuries or mental stress were discovered during this study. This indicates that the training volume and stress from the coaches did not affect the athletes. This also substantiates the argument of Slivka et al. that by limiting the stress on athletes from sources unrelated to training, athletes can withstand very high training volume and intensity without developing OTS [27].

POMS questionnaire and PSQI scale scores (measuring, e.g., sleep disorders and sleep latency) are considered early indicators of training maladaptation [26]. Studies have found that loss of sleep may have negative effects on subjective well-being measurements, including measurements of fatigue, mood, and confusion. Furthermore, competition stress and anxiety can negatively affect sleep quality and duration, and sleep loss can damage mood and increase stress and anxiety [26]. One study reported that athletes with lower self-assessed sleep quality have higher levels of emotional confusion, leading to a higher risk of failure. Each point increase in confusion level was discovered to lead to 19.7% lower sleep quality [27]. Hausswirth et al. assessed the POMS score and VO_2_max of 40 triathletes and continually monitored the participants using Actiwatch. Nine triathletes were diagnosed with functional overtraining with reduced functionality and high perceived fatigue [28]. A significant time–group interaction was also discovered between sleep duration, sleep efficiency, and inactive periods [28]. Romyn et al. also argued that greater tension and anxiety lead to lower sleep quality ratings [29]. Based on the studies above [26], the POMS and PSQI questionnaires can be used to evaluate early indicators of training maladaptation. However, observing only the changes in the total scores of these two questionnaires may neglect the detailed mental changes in these college triathletes. Likewise, our results revealed no significant differences in the total POMS and PSQI scores of athletes across training periods (Figure 4 and Figure 6). Importantly, we found that these athletes showed a significant increase in POMS stress levels during the competition period, yet there were no significant differences in their sleep quality PSQI scores among periods. Our obtained result seems to exhibit certain diversities from the above studies [27,28,29], and we speculate that these collegiate triathletes might have better attitude when controlling their life pattern, thereby exhibiting greater capacity to cope with mental stress of training or competition by obtaining better sleep quality.

Most studies have used the ESS, Competition Stressor Scale, Checklist for Sleep Hygiene, and PSQI scale to assess sleep and stress in athletes [15,16,30]. Mah et al. reported on inappropriate sleep times among collegiate athletes due to insufficient sleep hours and low sleep quality, and these athletes were often discovered to exhibit visible daytime sleepiness [15]. Reasons given in the literature for daytime sleepiness and poor sleep quality include high-intensity training, extra working time, overuse of electronic devices, skipping breakfast, sports injuries, overtraining, anxiety prior to sleep, and other problems that cause anxiety, pain, and sleep disorders [15,30]. However, it has to be noted that the ESS results in the present investigation revealed no significant differences between training months. The individual assessments revealed that although most of the athlete participants did not experience daytime sleepiness, some athletes had an average ESS score of 16–18 points, indicating that they experienced higher rates of daytime sleepiness. One study suggested that the training schedule prepared by sports team coaches can be personalized by being flexibly adjusted to the athlete’s needs and that practical guidance and suggestions given to athletes should lessen their perceived fatigue [17]. Based on the present findings, we suggest that collegiate triathlon coaches conduct individual interviews with athletes with higher sleepiness scores, change their training start time, or provide appropriate sleep-related health education to help these athletes reduce their daytime sleepiness.

Changes in athletes’ mental state or mood while training for super-endurance competitions can lead to changes in their performance [31]. Triathletes are considered a group at risk of developing a series of adverse health results due to high training intensity and long training times [18]. Additionally, mood is directly correlated with training amount and intensity [31]. Comotto et al. used sRPE and the POMS scale to monitor a group of teenage elite triathletes (age: 18 ± 1 years) who were given the same external training intensity during training camp. The POMS scores revealed that the athletes’ fatigue increased by 45% and vigor decreased by 24%, and the energy index for all training camp athletes decreased [32]. The POMS scores in a 6-month study on 32 triathletes (18 athletes, 15 members in the sedentary control group, all aged 24–61 years) discovered that vigor score decreased and anger and fatigue scores were high during peak training months [31]. Unlike prior studies that included participants with a wide age range, this study focused on collegiate triathletes and used the POMS scale to assess changes in their mood during 4 months of periodic training (this period included general physical preparations and national level competition stages). The results from the present study revealed that the fatigue scores among the collegiate triathletes were significantly higher when the training was most extensive; however, their anger scores were not significantly higher and their vigor scores were not significantly lower. Comparing the present findings with those of a previous study [31], we surmise that emotional stress was not significantly higher when training intensity was higher because of the similarities in age, periodic training schedule, and competition preparations among the collegiate triathletes. Furthermore, school sports teams have peer support, professional coach supervision, and team goals; these attributes could have helped to alleviate the stress caused by intensive training.

This study discovered that although the amount that collegiate triathletes trained differed depending on the particular training period, the differences did not significantly affect the athletes’ sleep quality or emotional response. This further substantiates the earlier statement that precompetition preparations that coincide with the winter holiday break allowed athletes to have sufficient rest despite an increase in training volume and greater depression, fatigue, and tension during the competition periods. These results show that collegiate triathletes have healthy emotion and sleep management and also that the reduction in academic pressure during the precompetition period may explain the athletes’ favorable sleep quality and mood. Although this study discovered no significant differences between training periods in the overall averages for sleep quality, daytime sleepiness, and emotional stress, some athletes did have significantly higher scores than other athletes in these portions of the questionnaire. These athletes may have had insufficient rest or recovery due to personal factors. Therefore, coaches should provide individualized guidance and recommendations to athletes whose scores indicate poor sleep quality or high stress or refer them to appropriate mental, nutritional, or sleep consultants.

### 4.1. Study Limitation

One of the primary limitations of the present study is that the participants were limited to national, college-level elite triathletes; therefore, all participants were trained and competed on the same college triathlon team. In this regard, we made every effort in the study design to eliminate potential confounding factors, including standardized dietary instruction by the team coach and the conducted supervised triathlon-specific training program. Despite our efforts to control the study design quality, we still could not completely rule out the possibility of a Type II error due to the relatively small sample size. However, the primary population in this study was collegiate Division I elite triathletes (all of them were ranked Top 10 in the nation in their competition category), which is a specific category and a very limited number of participants could be recruited. On the other hand, although both male and female athletes were included in this study; we did not further analyze the differences between males and females due to the small sample size. Therefore, future studies investigating gender differences in this issue are warranted.

### 4.2. Perspectives in Practical Application

The results of this study provide crucial information for triathlon training. (1) Questionnaire surveys (e.g., POMS and PSQI scales) have reliability and can reflect athletes’ physical and psychological status. Coaches should ask their athletes to regularly complete short personal surveys. (2) Triathlons are competitions for individuals, and different athletes have different physical and psychological statuses during training. Coaches must provide individualized instructions or adjustments based on the problems experienced by the particular athlete. (3) Coaches should consider student athletes’ academic stress state and training quality to individually adjust the training intensity and periodic program to achieve greater sports performance.

## 5. Conclusions

The emotional state, level of fatigue, and sleep quality of 13 collegiate triathletes going through periodic training were collected in this study and assessed over 3 months before the national competitive event. This study discovered that training volume was highest 1 month before a competition. The POMS overall emotional assessment scores indicated no significant differences between different training periods. However, anxiety scores rose during the competition season, while fatigue and depression scores rose during the peak training period. The personal fatigue scores indicated no significant changes in the fatigue level during the investigation period or any significantly greater fatigue during the peak training period. Changes in the ESS total score were also nonsignificant. The PSQI total score and individual scores were not significantly different, indicating favorable sleep quality among the athletes. A comprehensive view of the assessments used in this study—training volume, POMS, PSQI, ESS, and personal fatigue—indicate that the collegiate athletes exhibited healthy emotional and sleep states (PSQI score < 5) during each training period. The athletes had proficient self-discipline, time management, and mental adjustment skills. Administering a subjective questionnaire might help the coaches to closely consider each athlete’s individual demands. When selecting evaluation scales, a simply used questionnaire should be considered to minimize the inconvenience to athletes of completing forms after stressful studying and training intensity. Coaches should also appropriately intervene when the athletes exhibit negative patterns to help them better cope with psychological or physical stress to prevent overtraining.

## Figures and Tables

**Table 1:** Participants’ anthropometric profiles.

Measurement	Male (*n* = 8)	Female (*n* = 5)
Age (year)	20.0 ± 0.3	21.6 ± 0.7
Height (cm)	172.6 ± 1.8	158.4 ± 0.9
Weight (kg)	68.0 ± 2.5	56.7 ± 1.7
Body Mass Index (BMI, kg/m^2^)	22.8 ± 0.7	22.7 ± 0.8
Muscle mass (kg)	52.2 ± 1.5	37.9 ± 0.8
Body fat percentage (%)	19.6 ± 0.01	30.4 ± 0.01

## Data Availability

The data presented in this study are available upon request from the corresponding author.
